# Analysis of Genetic Diversity Based on Sequences of Feline Calicivirus Strains Isolated in China

**DOI:** 10.1155/tbed/9924540

**Published:** 2025-08-19

**Authors:** Yupeng Yang, Mengru Chen, Zhe Liu, Kexin Feng, Ruibin Qi, Hongtao Kang, Qian Jiang, Liandong Qu, Jiasen Liu

**Affiliations:** ^1^State Key Laboratory for Animal Disease Control and Prevention, Harbin Veterinary Research Institute, Chinese Academy of Agricultural Sciences, Harbin 150069, China; ^2^College of Veterinary Medicine, Northeast Agricultural University, Harbin 150030, China

**Keywords:** feline calicivirus, genetic diversity, genotypes, mutations, protein adaptability analysis, recombination, spatiotemporal evolution, VP1 protein

## Abstract

In recent years, feline calicivirus (FCV) has caused increasingly severe harm in China, posing a significant threat to feline health. However, our understanding of the complex epidemiology and genetic diversity of FCV in China remains limited. In this study, we integrated various bioinformatics methods and used isolates from China as the primary research subjects. The approach ranged from basic prevalence statistics to genome sequence analysis, systematic exploration of spatiotemporal evolution, recombination studies, comparisons of specific sites, structural biology predictions, protein adaptation analysis, and molecular dynamics simulations. This comprehensive approach aimed to obtain a thorough understanding of the characteristics of FCV in China. An in-depth analysis of the results indicates that FCV exhibits a nationwide epidemic trend in China mainly consisting of two genotypes: GI and GII. The prevalence rate of genotype GI exceeds 70%, making it the predominant epidemic genotype. Spatiotemporal evolution predicts that the ancestor of genotype GI emerged in 1821 while the ancestor of genotype GII emerged in 1879. After the 1950s, there was rapid expansion in the spread of FCV which extended from eastern parts to regions in southwest, southeast, and northeast after 1990. The analysis on recombinant evolution suggests that FCV can undergo recombination within same genotypes or across different genotypes enhancing its cross-species transmission and infectivity capabilities. Amino acid sequence analysis reveals mutations at key amino acid site position 481 involved in receptor binding where K mutated into E or N in domestic prevalent strains recently. All seven amino acid sites related virulence have undergone mutations. The results of protein adaptability analysis indicate that the amino acid residue at position 281 (N) in the VP1 protein is a site of adaptive selection. In some strains, the amino acid at this position has mutated from N to G, S, or R. Further molecular dynamics simulations reveal that these mutations affect the structural stability of the VP1 protein. The results of this study are essential for gaining a thorough understanding of the FCV profile in China and can be used to create better prevention and control strategies.

## 1. Introduction

Feline calicivirus (FCV) is one of the most widespread pathogens infecting felines in the world, causing mainly mild clinical signs and being a cause of respiratory disease in cats [1]. Since its initial discovery in New Zealand in 1957, there has been a steady increase in FCV cases globally. FCV has gained significant prevalence in numerous countries, particularly in recent years [2–8]. In the past few years, there have been reports of FCV outbreaks in various parts of China, such as the northeast [9], southwest [10], Guangdong [11], Beijing [12], Wuhan [13, 14], and Nanjing [15], among others. It is concerning that, apart from the classic strain, the highly pathogenic virulent systemic disease (VSD) strain, which results in cats dying due to systemic damage, has been observed not only in other countries, but also in China, and the VSD strain has become increasingly widespread worldwide in recent years [5, 12, 16–18]. Studies have demonstrated that FCV can affect a variety of cats, including tigers, cheetahs, and lions [19–21]. Astonishingly, there have been reports of dogs being carriers of FCV as well [22]. The VSD strain's ability to spread to cherished wildlife causes the demise of wild creatures, leading to massive financial losses. There is no doubt that the emergence of VSD strains has been a serious threat to feline health.

FCV, a member of the Caliciviridae family and Vesivirus genus, is an RNA virus characterized by its single-stranded positive strand and absence of an envelope [23]. Prior research has indicated that FCV possesses a single serotype while exhibiting two genotypes, with genotype II predominantly comprising Asian isolates [14, 24]. The division of FCV genotypes is associated with the *VP1* gene, and the virulence and antigenicity are also related to the *VP1* gene, which is divided into six regions, A, B, C, D, E, and F [25–27]. Among them, the E region has an extremely important role in the evolution of the virus, and it is also an important region for the study of FCV virulence and antigenicity [18, 25, 28–30]. Meanwhile, it was demonstrated that p30 is the primary gene responsible for the varying levels of virulence in FCV strains, which is critical for understanding FCV virulence [31]. It is worth noting that FCV, similar to other positive-stranded RNA viruses (e.g., poliovirus) [32, 33], also undergoes recombination, a phenomenon that has been documented by numerous scholars [10, 11, 34, 35], and a significant number of cats who have been clinically cured still harbor FCV [2], thereby, enhancing the likelihood of recombination. The possibility of recombination among various FCV strains should also be taken into consideration.

The high variability of FCV and the emergence of VSD strains have placed greater demands on its prevention, but existing vaccines are less effective in preventing the prevalence of the virus. Studies have shown the high transmissibility of FCV and the inability of vaccines to provide complete protection in China make it a significant threat to felines [13, 36]. In this study, we employed various bioinformatics techniques to conduct a deeper understanding of the genetic diversity, adaptive evolution, and transmission dynamics of FCV isolates in China. By understanding the current epidemiological status of FCV in China, accurately predicting its epidemic trends, and revealing its genetic variations and transmission evolution, in order to provide a more effective reference for vaccine prevention research.

## 2. Materials and Methods

### 2.1. The Prevalence of FCVs in China

We conducted a comprehensive analysis of the geographical, host, and temporal characteristics of FCV, utilizing FCV strains that were isolated and preserved in our laboratory, as well as all Chinese FCV isolates obtained from the National Center for Biotechnology Information (NCBI) (with a sequence collection up to 2021) (https://www.ncbi.nlm.nih.gov/).

### 2.2. Genome-Wide Analysis of FCV

The nucleotide sequences of publicly available whole genomes including all the FCV strains in China as well as the nucleotide sequences of foreign classical strains were retrieved from the NCBI. The whole-genome sequences that were downloaded underwent a comparative analysis for nucleotide sequences using MEGA-X software and were subsequently adjusted [37]. The entire set of nucleotide sequences was then employed to build a phylogenetic tree, comprising the traditional phylogenetic tree and the radiation phylogenetic tree, with the conditions outlined in the figure notes. Phylogenetic tree modification: https://www.chiplot.online/ [38]. Simultaneously, the homology of FCV genome-wide nucleotide sequences underwent additional investigation, while the identification of strains from diverse hosts and genotypes was analyzed and contrasted.

### 2.3. Sequence Analysis of FCV *VP1*

Nucleotide and amino acid sequences of *VP1* from 86 FCV strains were retrieved from NCBI. This dataset included *VP1* sequences extracted from all available Chinese whole-genome sequences, supplemented by representative sequences from classical foreign strains. The strains on NCBI were divided into sequences based on their specific collection time, with a time limit of 2021. Subsequently, the gathered sequences underwent processing and alignment of nucleotides and amino acids was executed utilizing MegAlign software. The complete comparison results were retained for phylogenetic analysis using MEGA-X software respectively. Phylogenetic tree modification: https://www.chiplot.online/ [38]. Meanwhile, the homology of FCV *VP1* amino acid sequences was further examined, and the strains from various hosts and genotypes were analyzed and contrasted with one another.

### 2.4. Evolutionary Dynamic Analysis

This study aimed to employ the *VP1* amino acid sequences of FCV for the purpose of spatiotemporal genetic evolutionary analysis, thereby, enhancing our comprehension of the epidemiology of FCV in China. Consequently, the BEAST software package (version 1.10.4) was utilized to estimate the time to the most recent common ancestor (tMRCA) and evolutionary rate based on the 86 *VP1* sequences mentioned earlier [39]. In order to guarantee accurate rate estimates in the temporal structure of *VP1* sequences, we initially examined and determined the sampling times for all strains, and subsequently employed the TempEst software [40] to regress apical distances and sampling times, thereby, eliminating any outlier sequences and guaranteeing the precision of the outcomes. To obtain more robust ratio estimates, we used the Bayesian Monte Carlo Markov Chain (MCMC) method implemented in the BEAST software package (version 1.10.4), incorporating a JTT nucleotide substitution model, Gamma + invariant Sites, number of gamma categories of 4, relaxed molecular clock model and coalescent: bayesian skyline tree prior. The analysis was run for a total of 50 million generations, with sampling every 5000 generations. Convergence of the parameters of interest (i.e., effective sample size greater than 200) was assessed using Tracer (version 1.10.4). After that, the BEAST software was used to obtain the posterior distribution of the tree (10% burn-in) and to obtain the Bayesian maximum clade credibility (MCC) tree [41]. The BEAST package was used to calculate tMRCAs. Ultimately, the time tree underwent analysis and landscaping utilizing FigTree (version 1.4.4).

### 2.5. Spatiotemporal Evolutionary Characterization of FCV

This study delved deeper into the spatiotemporal dynamics of FCV in China, specifically examining the estimation of the virus's ancestral location and its evolution over time using Bayesian discrete phylogeographic techniques, depicted through visual representations. The SpreaD3_v0.9.6 software was utilized to initially visualize the results, followed by the generation and storage of D3 files in a designated folder for the inferred migration routes and the most substantial spread rates [42]. Ultimately, the archived document was accessed on a webpage to generate an animated depiction of the spatiotemporal trajectory of FCV dispersion in China. To facilitate observation, critical time points are selected for interception and analysis, and planar two-dimensional graphs are created accordingly.

### 2.6. Recombination Analysis

Potential recombination events were analyzed using Simplot version 3.5.1 and RDP3 software. The RDP3 software mainly used methods such as RDP, GENECONV, Bootscan, Maxchi, Chimera, SiSscan, Phylpro, LARD, and 3Seq. Recombination was initially identified when the outcome of these methods was “+”. However, the recombination event was not initially recognized until a minimum of five of the employed methods were capable of detecting recombination. Using Simplot software, the recombinant strains were re-examined to ascertain if the recombination had taken place and to pinpoint the recombination site.

### 2.7. Examining the Major Amino Acids of the *VP1* Gene in Comparison

The WebLogo website (https://weblogo.berkeley.edu/logo.cgi) was utilized to compare and analyze the amino acid sequences of the *VP1* E region in FCV isolates from China. The *VP1* gene's amino acid sequences were subjected to statistical analysis, encompassing seven significant virulence-related sites and four receptor-binding-related sites, taking into account the diverse hosts, virulence, and genotypes of the strains.

### 2.8. Prediction of Positive Selection Sites

To further understand the trend of FCV genetic variation in China, the *p30* and *VP1* genes were used for positive site selection analyses. Four of the methods in the Datamonkey website (http://www.datamonkey.org/) were mainly used, including single likelihood ancestor counting (SLAC), fixed effects likelihood (FEL), mixed effects model of evolution (MEME), and fast unconstrained Bayesian AppRoximation (FUBAR) for inferring selection. SLAC, FEL, MEME, and FUBAR are among the techniques employed for inferring selection. The *p*-value threshold for SLAC, FEL, and MEME was set at 0.1, while FUBAR had a significance level of 0.9, and the loci detected by more than two algorithms were deemed to be positively selected. The MEGA-X software was utilized to compare and statistically analyze the amino acids of the positively selected loci, which were then presented in a tabular format. SWISS-MODEL (http://www.swissmodel.expasy.org/) was utilized to model the structures of p30 and VP1 proteins, while PyMOL (https://pymol.org/2/) was employed to modify the 3D structures of both proteins, in order to provide a visual representation of the study.

### 2.9. Molecular Dynamics Simulation

The predicted crystal structure of the F9 *VP1* gene was used as the backbone, and mutants were constructed by mutating the amino acids at the positive selection sites accordingly. The molecular dynamics simulation software GROMACS 2021 (https://doi.org/10.5281/zenodo.6801842) was utilized to conduct 30 ns kinetic simulations on the anticipated crystal structures of F9 and its associated mutants. The system was set at 312 K constant temperature and atmospheric pressure, and the force field was modeled using Amber14sb, explicit solvent model, LINCS constraints on hydrogen-atom related covalent bonding, and simulations were carried out independently for each mutant in steps of 1 fs and full length of 30 ns.

## 3. Results

### 3.1. The Prevalence of FCVs in China

**Figure 1:**
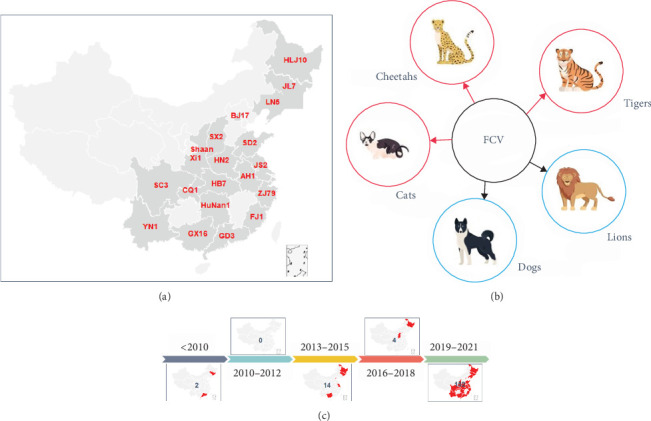
The prevalence of FCV in China is analyzed based on information downloaded from FCV in the NCBI. (a) Statistical maps of FCV in different provinces of China; (b) statistics of FCV infection in different hosts (red circles indicate reported FCV hosts in China, and blue circles represent hosts of FCVs not reported in China); (c) statistical results of FCV strains in China over different periods of time.

Statistical analysis was conducted on the background information of FCV strains registered in the NCBI (encompassing strains with only *VP1* sequences uploaded), along with the host, isolation time, and geographic region of each strain. The prevalence of FCV has been reported in more than 20 provinces, and the epidemic areas have covered most regions in China (Figure 1a). Additionally, the prevalent hosts of FCV in China are primarily cats, cheetahs, and tigers, while lions and dogs have not been reported domestically (Figure 1b). Temporal analysis reveals that since 2019, the reports of FCV prevalence in China have gradually increased (Figure 1c).

### 3.2. Phylogenetic Analysis of the FCV Genome

**Figure 2:**
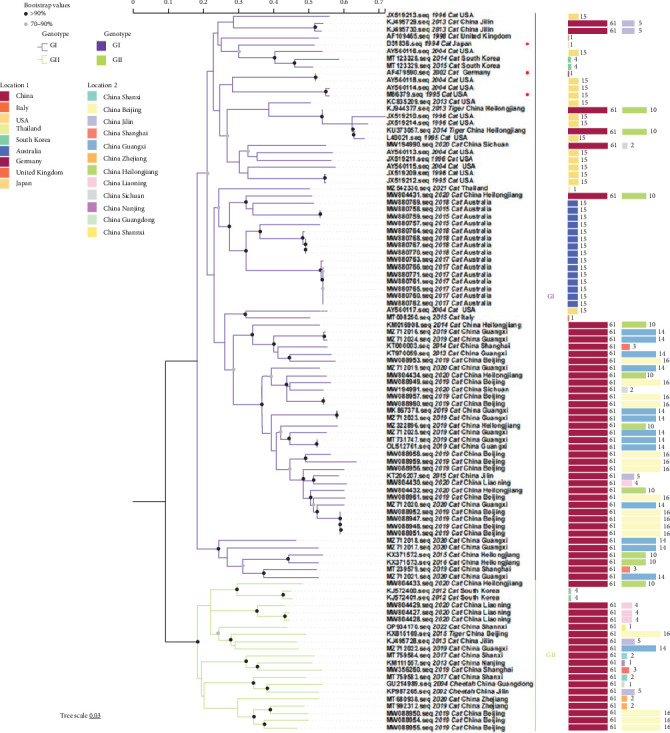
ML traditional phylogenetic tree estimated from 100 FCV genome nucleotide sequences including the sequences of strains isolated in our laboratory and those downloaded from the NCBI. Model: JTT + G + I; bootstrap: 1000 replicates. “·” represents the sequence used in this study. Each genotype is shaded by a different color and clearly marked; Location 1 on the right side of the phylogenetic tree represents the total number of isolates from each country, and Location 2 represents the total number of isolates from each province in China. Phylogenetic tree modification: https://www.chiplot.online/.

**Figure 3:**
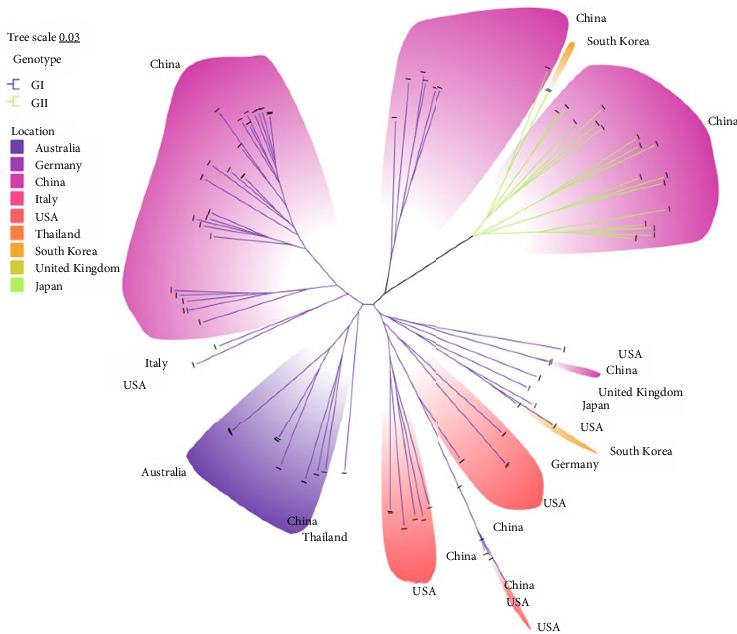
ML radiation phylogenetic tree estimated from 100 FCV genome nucleotide sequences including the sequences of strains isolated in our laboratory and those downloaded from the NCBI. Model: JTT + G + I; bootstrap: 1000 replicates. Strains from different countries are covered in different colors. Phylogenetic tree modification: https://www.chiplot.online/.

Genetic evolution analysis was performed based on an ML phylogenetic tree constructed from full-genome sequences of 61 domestic and 39 foreign FCV strains. The results showed that FCV could be classified into two genotypes: GI and GII. GI strains were isolated from regions such as the America, Europe, and Asia, while GII strains were only isolated from Asia. Among the currently prevalent strains in China, 43 strains belonged to genotype GI, accounting for 70.5% (43/61); 18 strains belonged to genotype GII, accounting for 29.5% (18/61) (Figures 2 and 3). This indicates that the prevalent strains in China are primarily of the GI strains. The results of the traditional phylogenetic tree analysis showed that the evolutionary divergence of FCV strains originating from the same region varied, and the genetic distance between FCV isolates in China and the three vaccine strains (F9, F4, and 255) was relatively large (Figure 2). The analysis of the radiation phylogenetic tree revealed that, despite the majority of Chinese isolates being in GI, there was a noticeable aggregation phenomenon, and combined with GII strains to form three significant branches (Figure 3). Subsequent nucleotide homology analysis revealed a range of 76.0% to 82.8% in the homology between Chinese strains situated in GI and GII, indicating a lower level of homology compared to strains of the identical genotype (Table S1). Significantly, the Chinese strains of GI or GII and F9 or 255 exhibited homology ranging from 78.0% to 79.7% (GI and F9), 76.0% to 77.9% (GI and 255), 78.5% to 79.9% (GII and F9), and 76.4% to 78.3% (GII and 255), respectively, all falling below 80%, and GII homology was even lower (Table S1).

### 3.3. Phylogenetic Analysis of the *VP1* Gene

**Figure 4:**
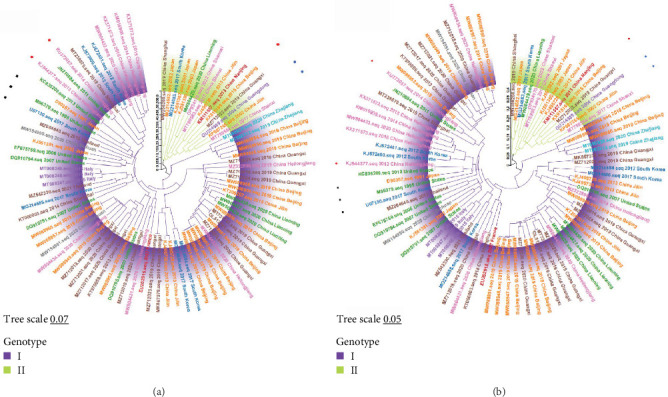
ML phylogenetic tree estimated from 86 FCV capsid gene (*VP1*) nucleotide sequences (a) and amino acid sequences (b) including the sequences of strains isolated in different countries. Model: JTT + G + I; bootstrap: 1000 replicates. The red and blue circles represent strains isolated from tigers and cheetahs, respectively, and the black squares represent the three vaccine strains. Each genotype is shaded by a different color and clearly marked. The scale bar indicates the mean number of nucleotide or amino acid substitutions per site. Phylogenetic tree modification: https://www.chiplot.online/.

ML phylogenetic tree was constructed based on the nucleotide and amino acid sequences of the FCV *VP1* gene from 86 strains (61 domestic strains and 25 foreign strains) for genetic evolution analysis (Figure 4). The results indicated that FCV could be classified into genotype GI and genotype GII. GI strains were isolated from regions such as America, Europe, and Asia, while GII strains were only isolated from Asia. Among the currently prevalent strains in China, 50 strains belonged to GI, accounting for 82.0% (50/61), and 11 strains belonged to GII, accounting for 18.0% (11/61). This suggests that the currently prevalent strains in China are primarily of the GI genotype. These results are consistent with the results of the phylogenetic tree constructed from the whole genome (Figure 4a,b). The three vaccine strains F4, F9, and 225 were genetically close to each other, but genetically distant from most of the Chinese isolates, which was also consistent with the results of the genome-wide constructed evolutionary trees (Figure 4a,b). Meanwhile, the sequence homology of amino acids in the *VP1* gene was collated, and there was no apparent difference in sequence homology between strains isolated from different hosts; the amino acid sequence homology between strains of type GI and strains of type GII was still lower than that between strains of the same genotype, and the sequence homology between strains of type GI and the vaccine strains of F9 and 225 was 82.5%−88.4% and 79.4%−88.1%, while sequence homology between GII strains and F9 and 225 vaccine strains was 83.2%–86.0% and 82.9%−83.9%, respectively (Table S2).

### 3.4. Evolutionary Dynamics of FCV in China

**Figure 5:**
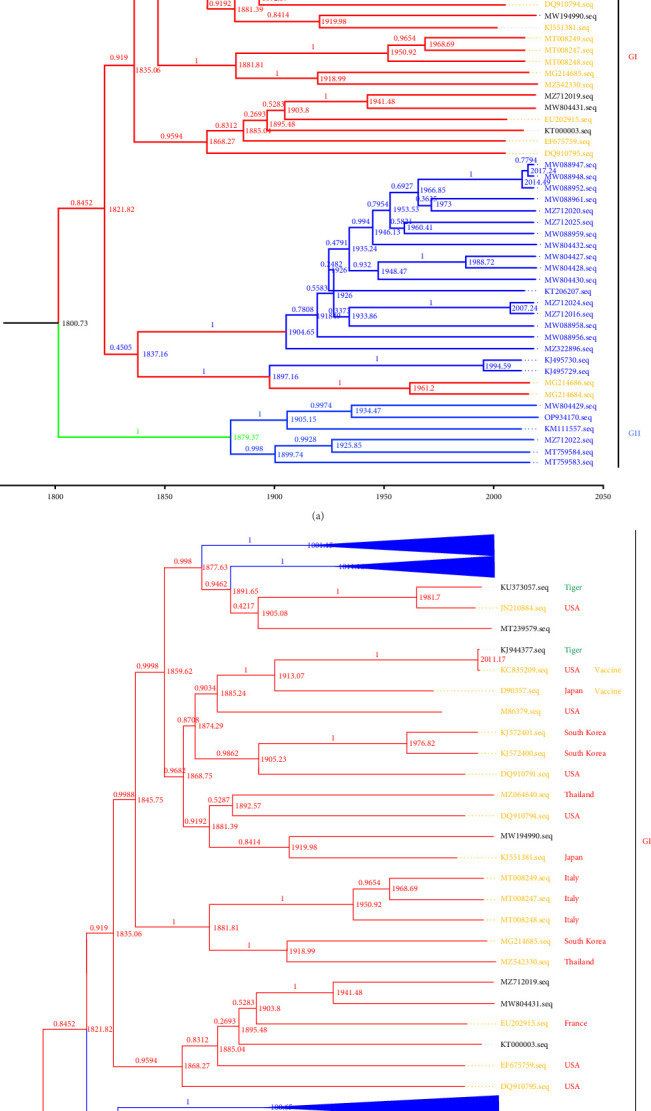
The MCC tree was reconstructed using BEAST (version 1.10.0). (a) The MCC tree was reconstructed; (b) The processing of Figure 5(a) allows a better analysis of the temporal evolution of FCV, the blue fold shows hidden domestic strains. The JTT + G + I distribution model and the coalescent: Bayesian skyline model with a total chain length of 5 × 10^7^ and sampled every 1000 times. Different colors of strain registration number markers represent strains from different countries, blue and black strain sequence numbers represent Chinese isolates, where large clusters can be clearly seen in blue; orange strain sequence numbers represent foreign isolates. Genotypes of FCV isolates are indicated with colored rectangular boxes.

In this study, the temporal signals in TempEst were first evaluated and found to be sufficiently strong (correlation coefficient = 0.6138, *R*^2^ = 0.3767), and amino acid sequences with subpar temporal signals temporal signals were removed, thus, facilitating the estimation of phylogenies for temporal calibration through the utilization of the molecular clock model. Based on the constructed maximum clade confidence (MCC) temporal tree, the analysis of the tree's genetic evolution shows that it closely resembles the ML tree derived from the *VP1* amino acid sequences mentioned earlier. The temporal analysis revealed that the most recent common ancestor of FCV could be traced back to the early 19^th^ century (Figure 5a,b). The emergence of different genotype ancestors varied, with genotype GI emerging around 1821 and genotype GII around 1879. The genetic diversity of the isolates from China started to rise and differentiate into various subtypes after 1850s. After 1950s, the virus population size rapidly expanded. Figure 5b provides a clearer depiction of the rapid growth and differentiation of the majority of representative strains from abroad in 1868, resulting in the formation of several distinct branches, which occurred much earlier than in China. Nevertheless, the analysis of the results presented in the time tree fails to establish a clear correlation between the dissemination of Chinese isolates and foreign isolates.

### 3.5. The Spread of FCV in China

**Figure 6:**
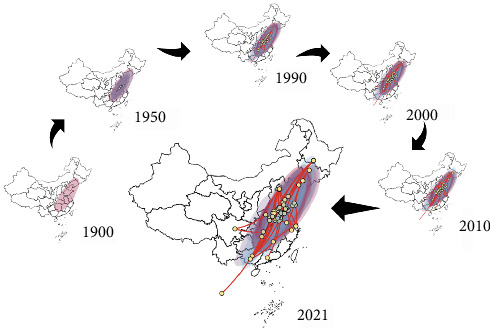
Distribution and spread of FCV in the China in the past few years. Discontinuous phylogeographic inference using a Bayesian system of amino acid sequences of the FCV VP1 protein to determine transmission and to select significant time points for interception.

The geographical distribution of Chinese strains (including one Thai strain) in the time tree was described using SpreadD3 software. Phylogeography was utilized to reconstruct geographic spread maps in an animated format, while planar 2D maps were created by intercepting images of significant time points. It was concluded that FCV started to emerge in eastern China in the early 1900s (Figure 6). Between 1900 and 1950, the spread of FCV was relatively slow. However, from 1950 to 1990, the rate of FCV dissemination accelerated, suggesting a potential rapid increase in its genetic diversity (Figure 6). It is evident that the transmission of FCV has become widespread throughout the country, including the areas of northeast, southwest, and southeast (Figure 6). It is certain that FCV will remain prevalent in China. A concern is that the lack of temporal and spatial analyses for FCV strains both domestically and internationally persists due to the absence of temporal continuity in FCV strains.

### 3.6. Recombination Analysis of FCV Whole Genome

**Figure 7:**
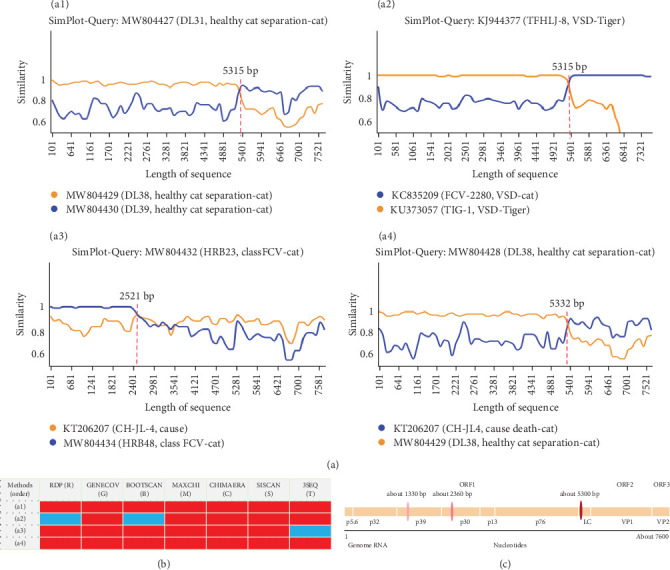
Analysis of the genome recombination profile of FCV. (a) Recombination between FCV strains of different virulence. (b) Recombination analysis of a1–a4 using different bioconfidence methods (red means recombination occurred and blue means no recombination occurred). (c) A display of the main recombination areas, with different color depths indicating the occurrence of different numbers of recombination events, with darker representing more events occurring.

Genome-wide recombination analysis of Chinese FCV isolates was performed using RDP4 and Simplot software, revealing potential recombination events with significant epidemiological implications, including the identification of recombinant strains and the search for recombination sites. The results showed that recombination is widespread in FCV populations and needs to be taken seriously. It was discovered in this research that strains MW804429 and MW804430 underwent recombination to form strain MW804427, marking a breakpoint at 5315 bp; the recombination of KC835209 and KU373057 resulted in the formation of strain KJ8944377, which serves as evidence of the recombination of two potent strains across species to create a strain capable of infecting large cats, such as tigers; it is worth mentioning that strain KT206207, which is capable of causing death in host cats, was recombined with the classical respiratory strain MW804434, forming a new classical disease-causing strain MW804432; and most interestingly the analysis showed that the lethal strain KT206207 and the asymptomatic strain MW804429 recombined to form the asymptomatic strain MW804428 (Figure 7a). Further analysis indicates that recombination can also occur between strains of different genotypes. For example, in the aforementioned recombination events, the GI strain MW804430 and the GII strain MW804429 exhibit distinct recombination signals, as do the GI strain KT206207 and the GII strain MW804429 (Figure 7a). RDP4 conducted a total of seven assays to further identify these four potential recombination phenomena, with over five confirming the occurrence of a recombination event, leaving no room for doubt that all four potential recombination events were once again demonstrated (Figure 7b). Two recombination software tools were employed to examine and tally the recombination sites, and multiple recombination sites were identified, with the most prone to recombination being around 5300 bp of the genome, followed by 2360 bp and 1330 bp (Figure 7c).

### 3.7. Comparative Results of Differences in Major Amino Acid Sites

Analysis of Chinese FCV isolates reveals critical variation patterns in the VP1 E region, highlighting distinct disparities in virulence-associated residues and receptor-binding sites. Comparative sequencs demonstrates exceptionally high genetic variation among strains, with the 5′ and 3′ regions being hypervariable and the central region remaining relatively conserved (Figure S1). Seven key virulence-linked amino acids exhibited host- and genotype-related mutations. Specifically, isolates from leopard and tiger origins aligned with three virulence-related sites (438, 440, and 445) of the VSD strains reported abroad. Chinese feline-origin VSD strains showed significant mutations, while non-VSD strains displayed high mutation frequencies—some converging on VSD-typical residues. GII strains novel acquired mutations at positions 452, 455, and 492 versus GI strains (Table 1). Given prior evidence implicating residues 444, 445, 480, and 481 in receptor binding, we profiled these sites against the F9 vaccine strain reference. Highly conserved 445D and 480K contrasted with recurrent mutations at positions 444 (mutated from N to S) and 481 (mutated to E and N) (Table 2).

### 3.8. Identification of FCV Adaptive Evolutionary Loci and Their Functional Analysis

To elucidate FCV evolutionary dynamics, we performed positive selection analysis on p30 and VP1 proteins from Chinese isolates. Integrated methodologies (Table 3) identified two positively selected sites: p30-T158 and VP1-N281. Structural mapping revealed p30-T158 resides in an α-helix, while VP1-N281 localizes to the Loop region of each trimer subunit (Figure S2). Site p30-158 is highly conserved, whereas VP1-281 exhibits partial mutation from ancestral N to S/G/R variants (Table 4).

**Figure 8:**
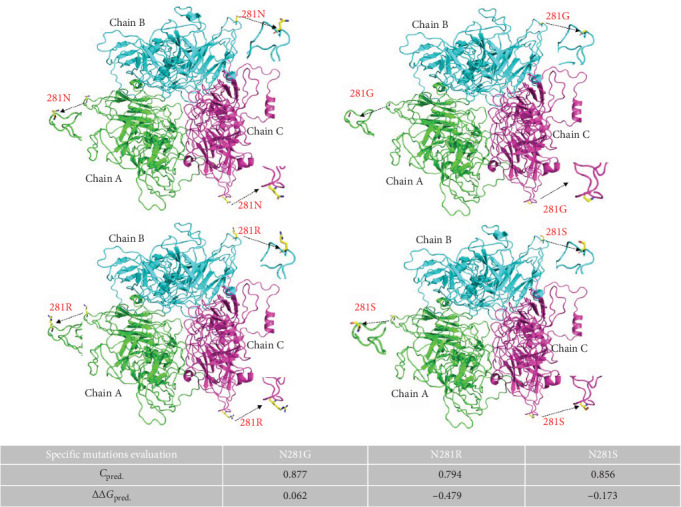
Prediction of the original and mutant trimer structure of F9 VP1 protein. Cartoon diagrams showing the trimeric structure of the VP1 protein were created using Pymol. Three single chains of the VP1 trimer are represented in green, blue, and pink, respectively. The adaptive amino acid site at Position 281 is represented by a sticks diagram with different colored atoms. Predictions of free energy changes are made below.

**Figure 9:**
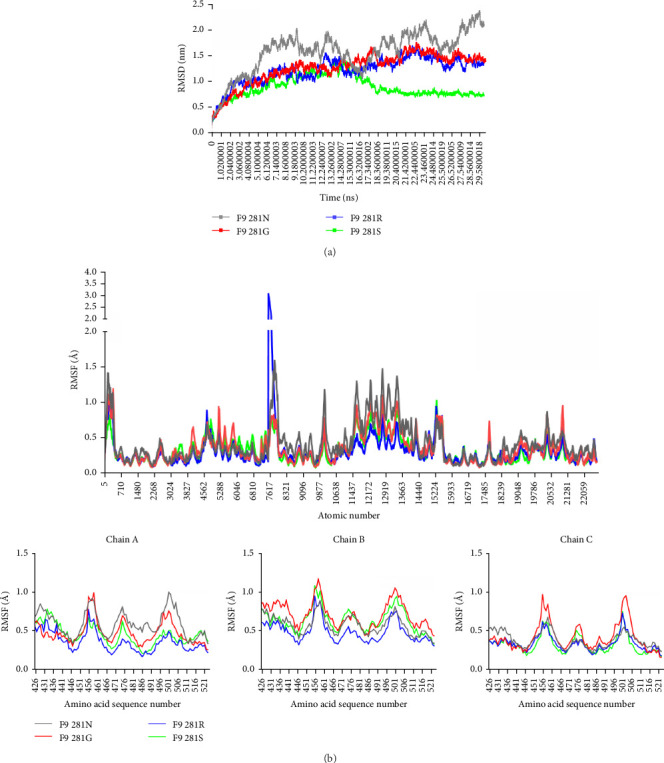
(a) RMSD of typical MD trajectories of the F9 VP1 protein original type and mutants (281). Solid lines of different colors represent different mutant phenotypes. (b) RMSF of the mutant (281) compared to the prototype. Solid lines of different colors represent different mutant phenotypes.

Given that specific mutations differentially impact viral architecture, we modeled VP1 mutants using the F9 vaccine strain as framework. Structural perturbations occurred at all mutant sites (Figure 8). Bioinformatics was further used to analyze the free energy of the mutant structures and molecular dynamics simulation was used to simulate the effect of different mutants on the structures, and to speculate the effect on the genetic evolution of the virus. The results obtained by calculating the difference free energies of the constructed 281G, 281S, and 281R mutants were 0.062, −0.173, and −0.479 respectively. The analysis of root-meansquare deviation (RMSD) showed that the three mutants 281G, 281S and 281R reached a steady state at 12, 16, and 19 ns, respectively, indicating that the structures had reached a stable state, with the fluctuations of 281G and 281R being less pronounced, whereas the initial structure of 281N exhibited greater fluctuations and did not attain a stable state (Figure 9a). The results of root-meansquare deviation (RMSF) were analyzed, and due to the existence of special structures, the N-terminal of each single strand with large fluctuations was excluded first (Figure S3); the amino acid region of 436–522 with large fluctuations in the results was mainly analyzed, which is also a crucial region associated with the virulence and antigenicity of FCV; overall, the mutant of 281G exhibited the highest fluctuation, the mutant of 281S displayed the second highest degree of fluctuation following the mutant of 281G, and the mutant of 281R had the lowest fluctuation (Figure 9b), and the lower fluctuation reflected a more stable structure. Above results were consistent with the predictions of free energy. The hydrophobicity of the mutated amino acids follows the order of G > S > R, suggesting that this effect may be related to hydrophobic forces. In summary, mutations at adaptive selection sites likely impact the stability of VP1 protein by altering hydrophobic forces in amino acids located at positions 436–522, potentially providing novel insights into the research of virulence mechanisms and vaccine development for FCV.

## 4. Discussion

The high mutability and genetic plasticity of FCV have created special challenges in diagnosis, prevention and treatment [1]. In China, there is a lack of systematic studies on the transmission and genetic evolution of FCV. Therefore, we pioneered the integrated application of multiomics approaches to delineate the epidemiological characteristics and genetic evolution of FCV in China, thereby, establishing a scientific foundation for rational vaccine design and targeted disease control strategies. Over the past decade, FCV infection rates have demonstrated persistent increases across China, with virologically confirmed cases documented in over 20 regions including Beijing, Shanghai, and Jiangsu, and so on. [10–15]. Preliminary epidemiological surveillance indicates transmission dynamics unconstrained by geographical barriers. Consequently, it is essential to conduct regular epidemiological research on FCV and provide a detailed overview.

Spiri et al. [3] have pointed out that the phylogenetic outcomes of FCV are hindered by sequence saturation, thus, necessitating the analysis of the complete sequences. In this study, we first analyzed the whole genome sequences of all FCVs for genetic evolution, followed by the *VP1* gene. Based on these ML tree results, we confirm that FCV can be classified into two distinct genotypes, with the strains exhibiting the GII genotype exclusively comprising Asian isolates, aligning with prior findings [14, 24]. The evolutionary tree revealed that despite the presence of Chinese strains in both GI and GII, they all exhibited distinct genetic subtypes, resulting in multiple aggregates. In China, the prevalence of FCV remains dominated by GI genotype, but the emergence of GII genotype may lead to more severe consequences. It is noteworthy that sequence homology comparisons of both the complete genome and *VP1* gene revealed substantial genetic distance between the Chinese-derived subtypes and three vaccine strains (F9, 255, and F4). This implies that current vaccines may result in immunological inefficacy against FCV. Our findings support the concept that existing vaccine strains potentially fail to provide comprehensive protection for felines [12, 43].

In addition to the genetic evolutionary analysis for the construction of ML evolutionary tree, we focused on the spatiotemporal evolutionary analysis, the study of the special loci of key genes, and the analysis of the evolutionary trend of gene selection, and so on, so as to comprehensively and systematically make predictions on the genetic evolution of FCV in China. The spatiotemporal evolutionary analysis of FCV employed Bayesian methods to reconstruct the VP1 amino acid-based MCC time tree. Through this approach, we estimated the ancestral emergence timeline of FCV in China, revealing divergent origins for GI and GII genotypes (19^th^ century for GI vs. early 20^th^ century for GII). These temporally distinct ancestry patterns indicate separate evolutionary lineages, offering critical insights for targeted FCV control strategies. Concurrently, the geospatial transmission model identified critical dissemination routes and high-risk hubs across China, providing evidence-based guidance for FCV epidemic containment.

Recombination analysis constitutes an essential methodology for investigating viral genetic evolution, particularly in RNA viruses, where recombination events frequently contribute to major outbreaks [44]. For instance, within the *Caliciviridae* family, norovirus recombinants pose elevated health risks to hosts [45, 46]. FCV recombination may pose potential threats, such as altered tissue tropism resulting from respiratory-enteric recombinant strains [34]. However, currently reported recombination analyses remain confined to single or limited isolates [10, 11, 35], lacking comprehensive and systematic recombination data across diverse viral strains. Our study expands upon existing findings, revealing significant results. Comprehensive recombination analysis demonstrates that recombination occurs between strains of distinct genotypes, virulence phenotypes, and host species origins, generating novel variants that facilitate adaptive viral evolution and cross-species transmission—closely matching recent reports of FCV recombinants infecting leopard cats (*Prionailurus bengalensis*) [47]. Subsequently, we identified three primary recombination breakpoints, with the most significant located at genomic position 5300 bp. The characterization of this recombination hotspot provides critical insights into recombination mechanisms. Collectively, the recombination analysis substantially advances our understanding of the FCV genetic evolution and informs evidence-based prevention strategies.

FCV exhibits extensive genetic variation, with certain strains evolving beyond conventional types into VSD variants capable of causing feline mortality. Tian et al. [31] successfully identified the nonstructural p30 protein as a critical determinant of FCV strain potency using strains F9 and 2280. Furthermore, existing literature demonstrates that the E-region of the VP1 protein significantly influences FCV pathogenicity and antigenicity [18, 29, 48]. Consequently, our investigation specifically analyzed key evolutionary sites within both p30 and VP1 proteins. The p30 protein demonstrates significant evolutionary conservation across FCV strains. In contrast, the *VP1* gene—notably its E-region—exhibits hypervariability. This study focuses on the VP1 protein E-region of Chinese FCV isolates, systematically analyzing amino acid variations at virulence-associated and receptor-binding sites. In VSD strains isolated domestically, virulence-associated sites exhibit significant mutations, with some displaying mutation patterns convergent with established VSD variants. Notably, a novel substitution mutation was detected at position 452 of genotype GII strains. Collectively, these findings suggest FCV may be evolving toward novel evolutionary trajectories, concurrently increasing the complexity of vaccine design. Subsequent investigations focused on fJAM-1, the primary cellular receptor for FCV entry. Prior studies have identified critical receptor-binding sites on the viral capsid [49–51], including cryo-EM structural characterization by Conley et al. [52] demonstrating high conservation of residues 444, 445, 480, and 481 during fJAM-1 engagement. However, we detected mutations at positions 444 and 481 (e.g., N444S and K481E) in circulating strains. Notably, the 481 site substitution may impair receptor-binding affinity, a futurer verification requiring functional validation through binding assays. Latest research indicates that the CDE region emerges as a key target antigen for subunit vaccines [53, 54]; systematic analysis of amino acid variations in the E region holds significant value in informing the development of next-generation vaccines.

Finally, we focused on analyzing amino acid mutation patterns, selective pressures, and adaptive evolutionary dynamics in the p30 and VP1 proteins, aiming to establish preliminary correlations between these variations and alterations in protein structure-function relationships. Among the identified positively selected sites (p30-158 and VP1-281), position VP1-281 exhibited high-frequency mutations in Chinese isolates. Using the VP1 protein of vaccine strain F9 as a structural scaffold, we successfully constructed 3D structural models of the mutants (281S, 281G, and 281R). Free energy analysis revealed significant differences: the 281R (ΔΔ*G*_pred_ = −0.479) and 281S (ΔΔ*G*_pred_ = −0.173) mutants exhibited negative free energy values, indicating substantially enhanced structural stability. In contrast, the 281G mutant showed increased free energy (ΔΔ*G*_pred_ = +0.062), suggesting reduced structural stability. Molecular dynamics simulations corroborated our hypotheses. RMSD and RMSF analyses demonstrated that the 281R mutant exhibited greater structural stability than the wild-type 281 N, while the stability of 281S and 281G progressively decreased. Notably, variations at this positively selected site may be intricately linked to molecular hydrophilic-hydrophobic balance. Hydrophobicity gradient analysis revealed the following order for residue 281: G > S > N > R. However, protein stability showed an inverse correlation: R > N > S > G. Interestingly, the alteration in the positive selection site may imply that the durability of the VP1 structure could be intricately connected to the hydrophobicity and hydrophilicity among the molecules, and that the hydrophobic forces significantly contribute to upholding the stability of the viral proteins and enabling protein interactions, and so on [55, 56]. The findings suggest hydrophobicity likely influences FCV capsid stability, and may provide valuable guidance for designing more stable versions of structure-based FCV vaccines (e.g., VLP vaccine).

In China, FCV is essential to increase surveillance as it has formed a nationwide epidemic due to multiple factors such as continuous mutation, widespread transmission, and cross-species infections. Our study offers a novel, systematic and more thorough insights into FCV epidemics in China. The aforementioned results provide a theoretical foundation and technical backing for the advancement of a more efficient FCV vaccine.

## 5. Conclusion

The results of this study show that FCV has become endemic in China, distinguished by two main genotypes, GI and GII. The prevalence of GI strains in China exceeds 70%, while the GII strains, all originating from Asia, account for less than 30%. Both genotypes display a significant genetic distance from the vaccine strains. The spatiotemporal analyses of the available sequences show that the ancestors of the GI and GII genotypes appeared at different times and that the eastern part of China is the key region for the spread of FCV. The recombination of FCV is more serious and produces different types of recombinant viruses with a variety of recombination sites. Some of the major amino acid sites in the VP1 protein have been mutated. The amino acid site 281 of VP1 being positively selected sites and mutated, potentially impacting the stability of the VP1 protein structure.

## Figures and Tables

**Table 1:** Comparison of amino acids at seven virulence factor-related sites between the Chinese isolates.

Strains	Amino acids at seven virulence factor-related sites						
	438	440	448	452	455	465	492
VSD strains (previous)	V/T	Q	K	E	T/D	S	V
ORD strains (previous)	T	G	A	D	D	G	V
Cheetah strains	V	Q	A/R	D	T/V	G	L
Tiger strains	T	S/Q	A/S	D	D/T	S/G	K/V/L
VSD strains	T	S/G/Q/D	A/L/R/K/S	D/E/N	D/T/S	S/G	L/V/I
Other strains	T/V/I/A	G/Q/N/R/K/S/E/P/D/L	A/P/G/R/K/S/H	D/E/A	D/T/S/W/G/L/I	S/G	L/V/I
GI strains	T/V/I/S/A	S/G/D/R/E/P/Q/L	A/P/K/S/L/R/G/H	D/E/N	D/G/S/N/L/I	S/G	V/I/K
GII strains	T/V	P/G/R/Q	R/A/P/G/S	D/E/A	D/T/V	S/G	L/I

**Table 2:** Comparison of amino acids at four receptor-related sites of Chinese isolates.

Strains	Amino acids at four major receptor-related sites			
	444	445	480	481
F9 (M86379)	N	D	K	K
Cheetah strains	N	D	K	K
Tiger strains	N_1_/S_2_	D	K	K
VSD strains (Feline)	N/S_1_	D	K	K/E_1_
Other strains (Feline)	N/S_7_/I_1_/D_1_/A_1_/G_1_	D	K	K/N_2_
GI strains	N/S_8_/I_1_/D_1_/A_1_/G_1_	D	K	K/E_1_/N_1_
GII strains	N/S_2_	D	K	K/N_1_

*Note:* The subscript indicates the frequency of occurrence of the amino acid.

**Table 3:** Positively selected sites in p30 and VP1 protein of FCV.

Proteins	Site	FUBAR (Post.Pro)	MEME (*p*-value)	FEL (*p*-value)	SLAC (*p*-value)
p30	158	0.938	0.01	0.0241	0.143
VP1	281	0.955	0.23	0.2053	0.0485

**Table 4:** Comparison across the adaptive evolution sites of the FCV p30 and VP1 protein with different type groups.

Strains	Cheetah strains	Tiger strains	VSD strains (feline)	Other strains (feline)	GI strains	GII strains
p30 (158)	T	T	T	T	T	T
VP1 (281)	S/G	S/N	N/S/R	N/S/G	N/S/G/R	N/S/G

## Data Availability

The data that support the findings of this study are available from the corresponding author upon reasonable request.

## Supporting Information

Supporting Information Table S1. Homology analysis of the whole genome sequence of FCV strains isolated from China. Table S2. Homology analysis of the VP1 amino acid sequence of FCV strains isolated from China. Figure S1. Amino acid comparison of capsid protein E region in FCV isolates from different regions of China. Each amino acid is represented by a different color, and the size of the graphic character is proportional to the frequency of occurrence of the amino acid at this locus. Figure S2. Cartoon diagrams were made using PyMOL software to show the structure of the positive selection site of p30 protein and VP1 protein, with blue, fuchsia, and orange colors indicating the helix, sheet, and loop regions, respectively. (a) Protein labeling diagram of the p30 protein positive selection site at position 157 (158) with different-colored atoms. (b) Protein labeling map of the positive selection site at position 281 of the VP1 protein with different colored atoms. Figure S3. Structural analysis of the N-terminal end of the three main chains of the FCV VP1 protein trimer.
