# Supplementary material for: Analysis of Genetic Diversity Based on Sequences of Feline Calicivirus Strains Isolated in China
Source: Transbound Emerg Dis. 2025 Aug 19;2025:9924540. doi: 10.1155/tbed/9924540 (PMC12380517; doi:10.1155/tbed/9924540)

**Supplementary materials**

**Table S1 Homology analysis of the whole genome sequence of FCV strains isolated from China**

**Table S2 Homology analysis of the VP1 amino acid sequence of FCV strains isolated from China**

**Figure S1. Amino acid comparison of capsid protein E region in FCV isolates from different regions of China. Each amino acid is represented by a different color, and the size of the graphic character is proportional to the frequency of occurrence of the amino acid at this locus.**

**Figure S2. Cartoon diagrams were made using PyMOL software to show the structure of the positive selection site of p30 protein and VP1 protein, with blue, fuchsia, and orange colors indicating the Helix, Sheet, and Loop regions, respectively.** (a) Protein labeling diagram of the p30 protein positive selection site at position 157(158) with different colored atoms. (b) Protein labeling map of the positive selection site at position 281 of the VP1 protein with different colored atoms.

**Figure S3. Structural analysis of the N-terminal end of the three main chains of the FCV VP1 protein trimer.**

**Table S1**

| Strains | FCV genomes(nn) | | | | |
| --- | --- | --- | --- | --- | --- |
|  | Cheetah strains | Tiger  strains | Feline  strains | GI  strains | GII  strains |
| Cheetah strains | 84.3% |  |  |  |  |
| Tiger  strains | 76.1%-  79.8% | 76.6%-  93.6% |  |  |  |
| Feline  strains | 75.9%-  83.8% | 76.1%-  82.8% | 76.0%-  100% |  |  |
| GI  strains | 75.6%-  78.6% | 76.1%-  80.5 | 76.1%-  89.0% | 78.4%-  97.9% |  |
| GII  strains | 78.0%-  83.8% | 76.1%-  82.8% | 76.0%-  82.5% | 76.0%-  82.8% | 78.1%-  100% |
| F9 strain  (M86379) | 75.4%-  75.8% | 76.3%-  79.6% | 76.1%-  79.7% | 78.0%-  79.7% | 76.0%-  77.9% |
| 255 strain  (U07130) | 76.2%-  76.8% | 77.5%-  80.1% | 76.4%-  79.9% | 78.5%-  79.9% | 76.4%-  78.3% |

**Table S2**

| Strains | FCV ORF2(aa) | | | | |
| --- | --- | --- | --- | --- | --- |
|  | Cheetah strains | Tiger  strains | Feline  strains | GI  strains | GII  strains |
| Cheetah strains | 91.6% |  |  |  |  |
| Tiger  strains | 83.5%-  90.9% | 83.7%-  87.5% |  |  |  |
| Feline  strains | 82.5%-  91.6% | 80.7%-  90.9% | 81.1%-  100% |  |  |
| GI  strains | 82.5%-  85% | 80.7%-  90.9% | 80.6%-  94.7% | 81.7%-  90.3% |  |
| GII  strains | 86.8%-  91.8% | 82.2%-  90.9% | 81.1%-  91.6% | 81.1%-  86.3% | 81.4%-  99.7% |
| F9 strain  (M86379) | 84.2%-  85.0% | 84.7%-  88.6% | 82.5%-  88.4% | 82.5%-  88.4% | 83.2%-  86.0% |
| 255 strain  (U07130) | 84.1%-  84.7% | 84.7%-  87.6% | 79.4%-  88.1% | 79.4%-  88.1% | 82.9%-  83.9% |

**Figure S1.**


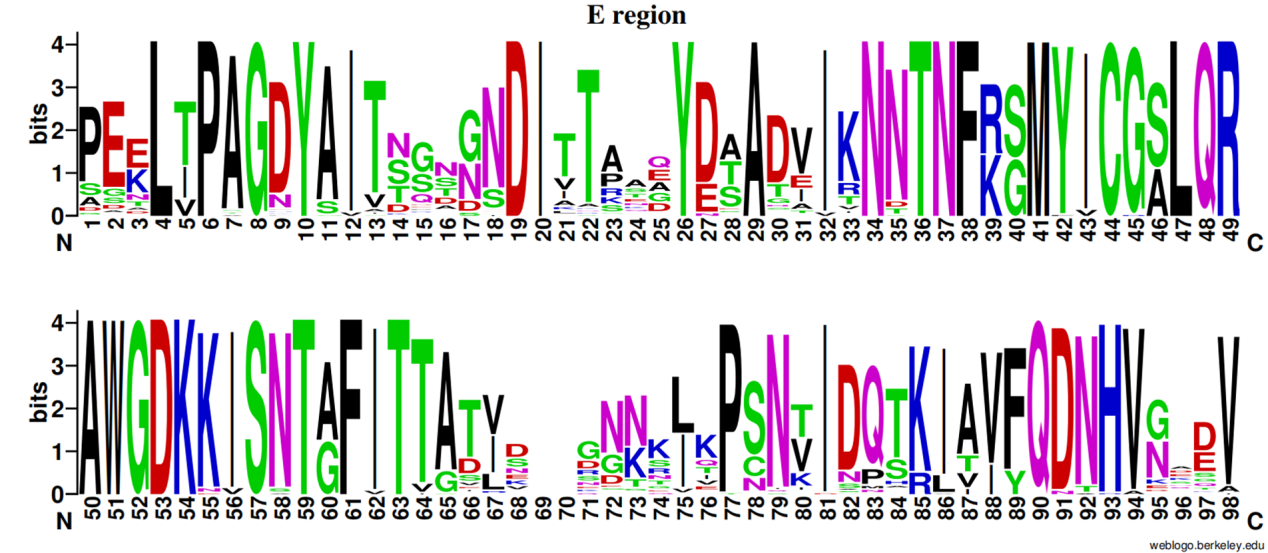


**Figure S2.**


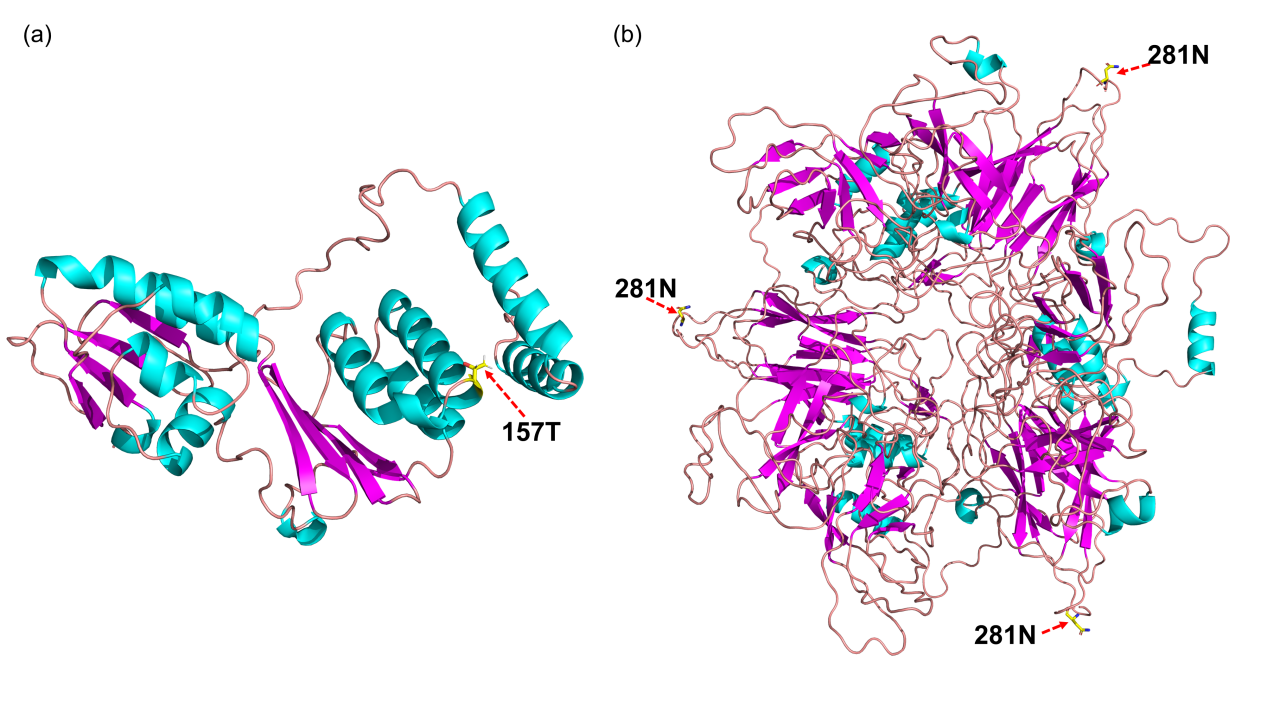


**Figure S3.**


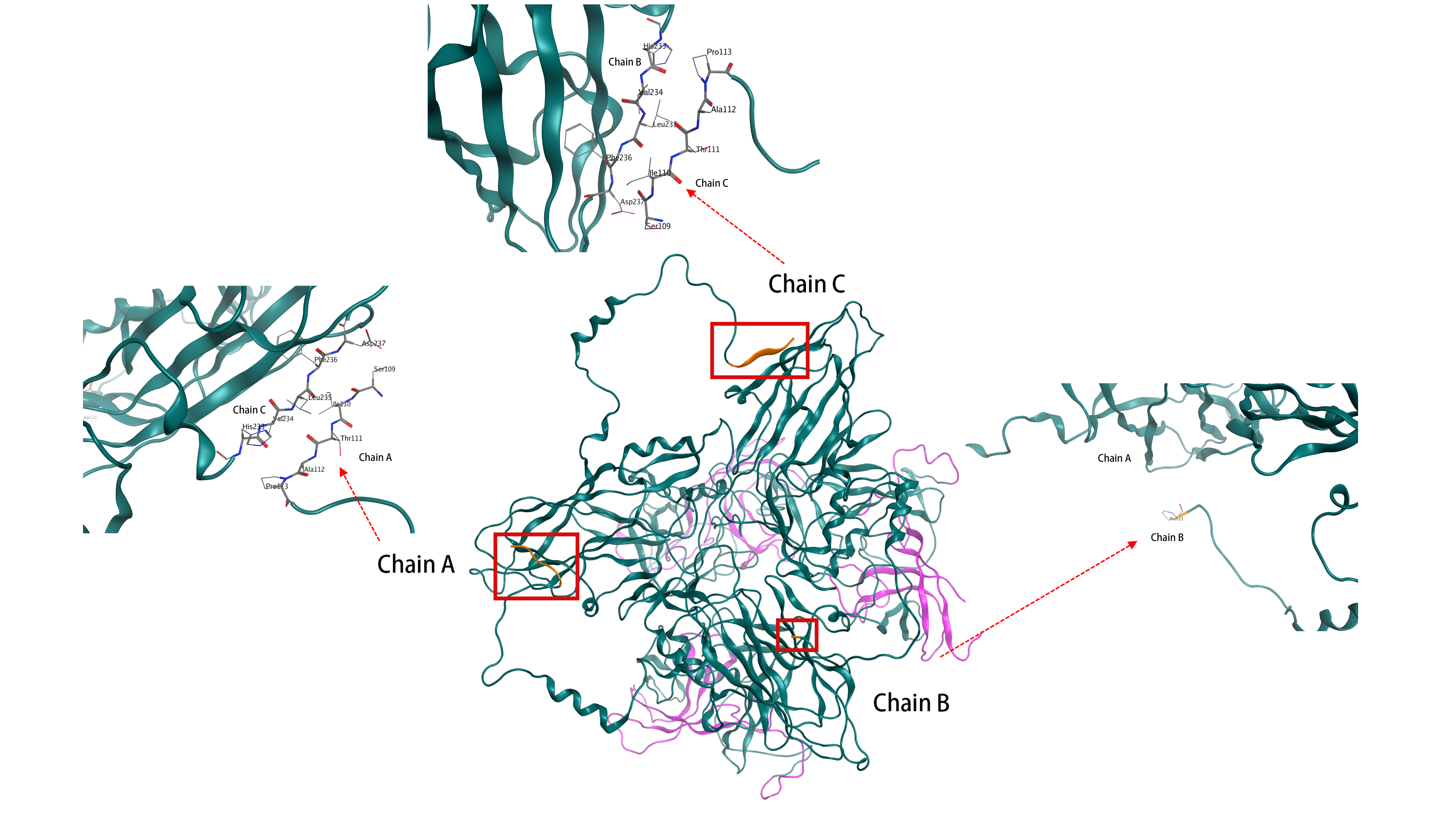

Supplement: Supporting Information — Table S1. Homology analysis of the whole genome sequence of FCV strains isolated from China. Table S2. Homology analysis of the VP1 amino acid sequence of FCV strains isolated from China. Figure S1. Amino acid comparison of capsid protein E region in FCV isolates from different regions of China. Each amino acid is represented by a different color, and the size of the graphic character is proportional to the frequency of occurrence of the amino acid at this locus. Figure S2. Cartoon diagrams were made using PyMOL software to show the structure of the positive selection site of p30 protein and VP1 protein, with blue, fuchsia, and orange colors indicating the helix, sheet, and loop regions, respectively. (a) Protein labeling diagram of the p30 protein positive selection site at position 157 (158) with different-colored atoms. (b) Protein labeling map of the positive selection site at position 281 of the VP1 protein with different colored atoms. Figure S3. Structural analysis of the N-terminal end of the three main chains of the FCV VP1 protein trimer. [file 9924540.f1.docx]
